# Clinical Metagenomic Sequencing for Species Identification and Antimicrobial Resistance Prediction in Orthopedic Device Infection

**DOI:** 10.1128/jcm.02156-21

**Published:** 2022-03-31

**Authors:** Teresa L. Street, Nicholas D. Sanderson, Camille Kolenda, James Kavanagh, Hayleah Pickford, Sarah Hoosdally, Jack Cregan, Carol Taunt, Emma Jones, Sarah Oakley, Bridget L. Atkins, Maria Dudareva, Martin A. McNally, Justin O’Grady, Derrick W. Crook, David W. Eyre

**Affiliations:** a Nuffield Department of Medicine, University of Oxford, John Radcliffe Hospital, Oxford, United Kingdom; b NIHR Oxford Biomedical Research Centre, John Radcliffe Hospital, Oxford, United Kingdom; c Department of Bacteriology, Lyon University Hospital, Lyon, France; d Microbiology Laboratory, John Radcliffe Hospital, Oxford University Hospitals NHS Foundation Trust, Oxford, United Kingdom; e Bone Infection Unit, Nuffield Orthopaedic Centre, Oxford University Hospitals NHS Foundation Trust, Oxford, United Kingdom; f Quadram Institute Bioscience, Norwich, United Kingdom; g Big Data Institute, Nuffield Department of Population Health, University of Oxford, Oxford, United Kingdom; National Institute of Allergy and Infectious Diseases

**Keywords:** orthopedic device infection, metagenomics, host depletion, antimicrobial resistance

## Abstract

Diagnosis of orthopedic device-related infection is challenging, and causative pathogens may be difficult to culture. Metagenomic sequencing can diagnose infections without culture, but attempts to detect antimicrobial resistance (AMR) determinants using metagenomic data have been less successful. Human DNA depletion may maximize the amount of microbial DNA sequence data available for analysis. Human DNA depletion by saponin was tested in 115 sonication fluid samples generated following revision arthroplasty surgery, comprising 67 where pathogens were detected by culture and 48 culture-negative samples. Metagenomic sequencing was performed on the Oxford Nanopore Technologies GridION platform. Filtering thresholds for detection of true species versus contamination or taxonomic misclassification were determined. Mobile and chromosomal genetic AMR determinants were identified in Staphylococcus aureus-positive samples. Of 114 samples generating sequence data, species-level positive percent agreement between metagenomic sequencing and culture was 50/65 (77%; 95% confidence interval [CI], 65 to 86%) and negative percent agreement was 103/114 (90%; 95% CI, 83 to 95%). Saponin treatment reduced the proportion of human bases sequenced in comparison to 5-μm filtration from a median (interquartile range [IQR]) of 98.1% (87.0% to 99.9%) to 11.9% (0.4% to 67.0%), improving reference genome coverage at a 10-fold depth from 18.7% (0.30% to 85.7%) to 84.3% (12.9% to 93.8%). Metagenomic sequencing predicted 13/15 (87%) resistant and 74/74 (100%) susceptible phenotypes where sufficient data were available for analysis. Metagenomic nanopore sequencing coupled with human DNA depletion has the potential to detect AMR in addition to species detection in orthopedic device-related infection. Further work is required to develop pathogen-agnostic human DNA depletion methods, improving AMR determinant detection and allowing its application to other infection types.

## INTRODUCTION

Infection is a serious and challenging complication of orthopedic-implant surgery, occurring in up to 2% of joint replacement procedures (1, 2). It may result in multiple surgeries and long-term antimicrobial treatment, with significant impacts on patient well-being and health care costs (1–3). The current gold standard for diagnosis of prosthetic joint infection (PJI) is culture of multiple periprosthetic tissue (PPT) samples (4–6), although this is slow and relatively insensitive, identifying a causative organism in as few as 65% of cases (4, 7). Sonication fluid culture may improve sensitivity (8) and is used alongside PPT culture in some centers (7). More comprehensive and rapid PJI diagnostics would potentially allow earlier and more targeted treatment.

Several studies (9–15) have shown metagenomic sequencing can identify causative pathogens in PJI, achieving species-level sensitivity of 83 to 96%, and potentially identifying difficult-to-grow organisms and pathogens following prior antibiotics. However, lack of comprehensive antimicrobial susceptibility prediction from orthopedic metagenomic sequencing has limited its application. We (16) and other authors (17–19) have applied long-read sequencing to link antimicrobial resistance (AMR) determinants to individual species within metagenomic samples. However, these approaches require extraction of sufficient pathogen DNA from clinical samples, which is often difficult given the overwhelming amount of human DNA frequently present. Without improved pathogen DNA yields, only species identification is possible and AMR prediction remains challenging.

We built on previous proof-of-principle work applying Oxford Nanopore sequencing for the diagnosis of PJI from sonication fluid (12), to evaluate a laboratory protocol for human DNA removal using saponin, a detergent previously demonstrated to differentially lyse human cells by disrupting the cell membrane (17, 20), and assess its impact on metagenomic sequencing-based AMR identification, specifically in Staphylococcus aureus as an example organism frequently causing PJI.

## MATERIALS AND METHODS

### Sample collection and processing.

Samples were collected at the Nuffield Orthopaedic Centre (NOC), a specialist musculoskeletal hospital with a dedicated bone infection unit within Oxford University Hospitals (OUH). Samples collected intraoperatively from revision arthroplasty surgery undertaken for suspected infection and aseptic failure between 15 June 2018 and 22 January 2020 were obtained following routine diagnostic workup. Ethical permission for use of the samples and linked deidentified metadata was granted by an NHS research ethics committee (reference 17/LO/1420).

Sonication fluids were generated from explanted prosthetic joints and other metalwork. Aerobic and anaerobic culture of sonication fluid and periprosthetic tissue (PPT) samples was performed as previously described (10, 21). Cultured organisms were identified using matrix-assisted laser desorption ionization–time of flight mass spectrometry (MALDI-TOF MS) (Bruker, Coventry, UK). Antimicrobial susceptibility of cultured bacteria was performed according to EUCAST methods either on a BD Phoenix 100 automated microbiology system (Becton Dickinson, Wokingham, UK) or manually by disc diffusion. Tissue samples also underwent histological examination.

### Sample preparation, sequencing, and data analysis.

Details of sample preparation (including human DNA removal with saponin, DNA extraction, library preparation), nanopore sequencing, metagenomic data processing and analysis, and thresholds for taxonomic classification are provided in the supplemental material. We compared species detection using metagenomic sequencing to culture results. As culture is potentially an imperfect reference standard, rather than reporting sensitivity and specificity, we report results in terms of positive percent agreement (PPA) and negative percent agreement (NPA), respectively (22). When evaluating the NPA, two approaches were used, one where species present in the sonication fluid cultures were used as the reference standard and one where species present in PPT cultures but not sonication fluid cultures were not considered false-positive results. For PPA calculations, we used sonication fluid cultures as the only reference standard, as for anatomical reasons it is possible that some species identified in PPT cultures were never present in the sonication fluid and therefore could not be sequenced.

### Detecting S. aureus AMR determinants.

AMR determinants were detected using methods previously described, requiring a minimum coverage depth of 20-fold for each resistance-conferring single nucleotide polymorphism (SNP) and 20-fold depth plus 100% coverage breadth for AMR genes on mobile genetic elements (16). Briefly, chromosomal variants were called after aligning reads to a reference genome (MRSA252) using minimap2 (23) (version 2.17-r941). Variants identified using Clair (24) (git commit 54c7dd4) were filtered with a trained random-forest classifier described previously (16) and then compared to a database of resistance-conferring SNPs (25) (see Table S1 in the supplemental material). Mobile genetic elements containing AMR genes were detected by minimap2 overlaps with a catalogue of genes adapted from previous studies (25) (Table S2). Detected genes, trimmed of surrounding sequence, were realigned to the resistance gene reference sequence.

### Data availability.

Sequencing reads classified as nonhuman for each sample have been deposited in the European Nucleotide Archive (PRJEB42910). The Nextflow (26) workflow used is provided at https://gitlab.com/ModernisingMedicalMicrobiology/genericbugontworkflow.

## RESULTS

One hundred fifteen sonication fluid samples from 113 patients underwent culture and metagenomic sequencing (Table S3). Forty-nine (43%) were culture positive for one (*n* = 48) or two (*n* = 1) organisms at >50 CFU/mL, 10 (9%) had <50 CFU/mL (6/10 with a highly pathogenic organism), and 8 (7%) were culture positive but not quantified (including one sample with three organisms isolated). Forty-eight (42%) samples were culture negative, of which 13 (27%) had evidence of acute inflammation on histology. Staphylococcal species were most commonly isolated, with Staphylococcus aureus accounting for 15/60 (25%) of all species cultured at >50 CFU/mL in sonication fluids (Table 1). Between 2 and 8 samples were run per sequencing flow cell using 52 flow cells overall, with a median sequence yield per flow cell of 10.2 Gb (interquartile range [IQR], 4.7 to 13.9). Efficient demultiplexing was achieved, with a median of 97% (IQR, 94 to 98%) of sequence bases assigned to a sample.

**TABLE 1:** Summary of species observed in culture for sonication fluid and PPT for 114 samples successfully sequenced^*a*^

Species	No. culture positive, sonication fluid	% Detected correctly by sequencing	No. culture-positive, PPT
Staphylococci	35	89	53
Staphylococcus aureus	19^*b*^	89	22
Staphylococcus capitis	1	100	3
Staphylococcus epidermidis	10	90	17
Staphylococcus haemolyticus	0		1
Staphylococcus lugdunensis	2	100	3
Other coagulase-negative staphylococci	3	67	7

Streptococci	8	100	12
Streptococcus agalactiae	1	100	2
Streptococcus dysgalactiae	4	100	6
Streptococcus oralis	2	100	3
Streptococcus pyogenes	1	100	1

Enterococci	4	50	8
Enterococcus faecalis	2	0	4
Enterococcus faecium	2	100	4

*Enterobacteriaceae*	9	67^*d*^	11
Enterobacter cloacae	4	50	5
Enterobacter species	1^*c*^	100	0
Escherichia coli	2	50	2
Klebsiella oxytoca	0		2
Proteus mirabilis	2^*c*^	0	2

Other	9	56^*d*^	20
Aspergillus species	0		1
Bacillus circulans	0		1
* Bacillus* species	0		2
* Candida* species	1	0	1
Cutibacterium acnes	2	100	4
Dermabacter hominis	1	0	1
Finegoldia magna	0		2
* Fusobacterium* species	1	0	1
* Paenibacillus* species	0		1
Parvimonas micra	1	100	0
* Peptoniphilus* species	0		1
Pseudomonas aeruginosa	3	33	4
Stenotrophomonas maltophilia	0		1

Total samples with no growth	52^*f*^		33^*e*^

a Results are reported where ≥1 isolate of a species was cultured. Sonication fluids were considered culture positive if >50 CFU/mL was isolated or if <50 CFU/mL of a pathogenic organism (i.e., not skin flora) was isolated and negative if no growth or <50 CFU/mL of an organism was isolated.

b Includes 4 samples where S. aureus was isolated at <50 CFU/mL.

c Includes one sample where the indicated species was isolated at <50 CFU/mL.

d Includes one species detected by sequencing to genus level only.

e Includes one patient where no tissue samples were collected during surgery.

f Includes samples with both no growth (*n* = 48) and <50 CFU/mL of a commensal organism isolated (*n* = 4).

### Effect of saponin on human and bacterial sequence yields.

**FIG 1:**
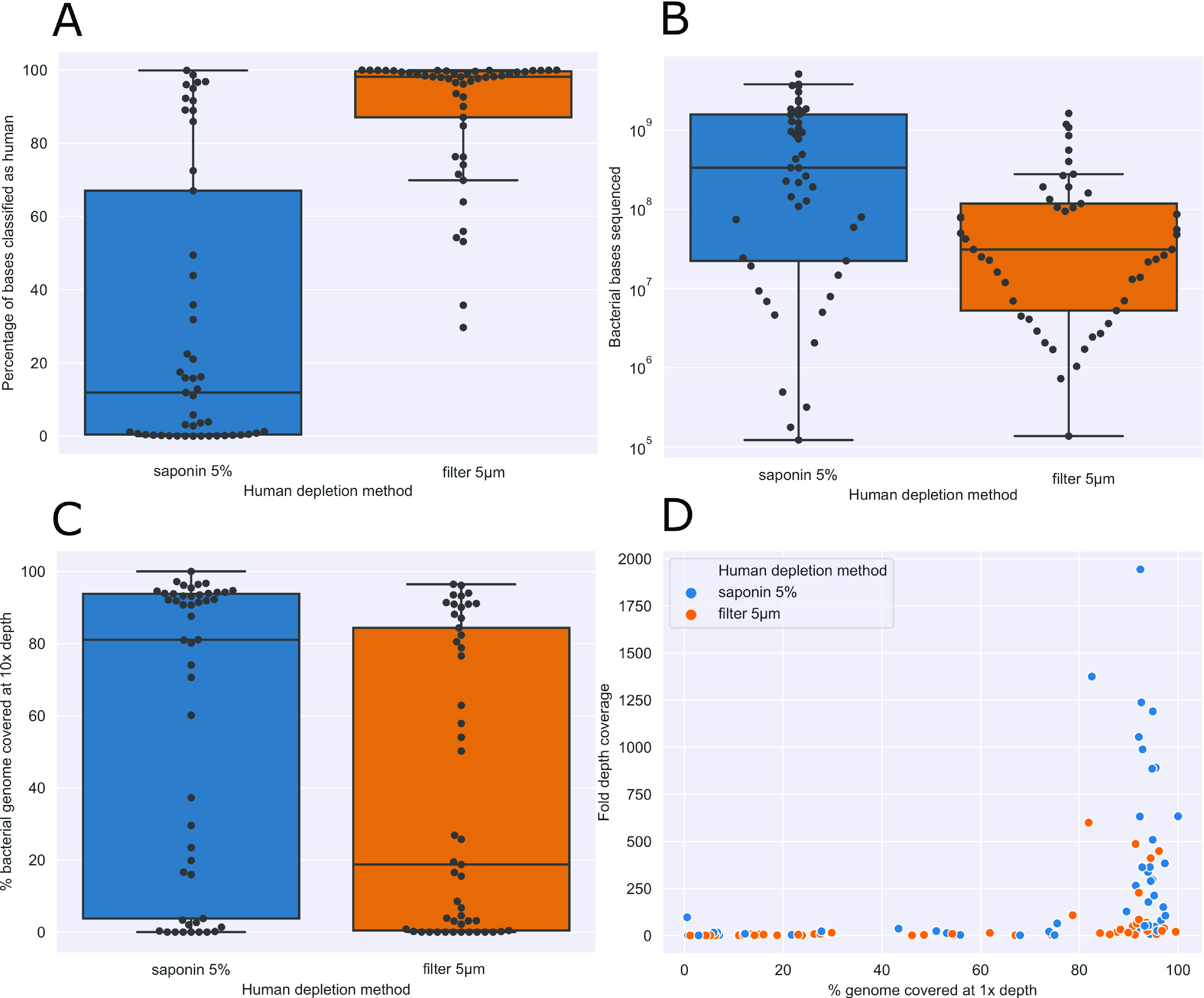
Effect of saponin treatment on human DNA depletion. (A) Proportion of bases sequenced classified as human, by treatment used. Statistical significance determined using a paired Wilcoxon signed-rank test with a *P* value of 1.11 × 10^−9^. (B) Number of bacterial bases sequenced, by treatment used. (C and D) Effect of saponin treatment on reference genome mapping breadth (C) and depth (D).

We compared human cell DNA depletion by saponin treatment to 5-μm filtration prior to DNA extraction. Ninety-one sonication fluid samples had sufficient volume (≥80 mL) to compare both depletion methods, of which 49 were culture positive. Treatment with 5% saponin reduced the proportion of human bases sequenced compared to 5-μm filtration (Fig. 1A), from a median (IQR) of 98.1% (87.0% to 99.9%) to 11.9% (0.4% to 67.0%) (Wilcoxon *P* < 0.001). A >80% reduction in human bases was observed in 30/49 samples (Table S4). There was a corresponding increase in median bacterial bases sequenced from 3.1 × 10^7^ (IQR, 5.3 × 10^6^ to 1.2 × 10^8^) to 3.3 × 10^8^ (2.2 × 10^7^ to 1.6 × 10^9^) (Fig. 1B). Comparing the proportion of reference genome covered at 10-fold depth (indicative of sufficient data for AMR prediction), saponin treatment increased the median (IQR) from 18.7% (0.30% to 85.7%) to 84.3% (12.9% to 93.8%) (Fig. 1C), as well as increasing genome coverage depth (Fig. 1D).

### Species detection.

**FIG 2:**
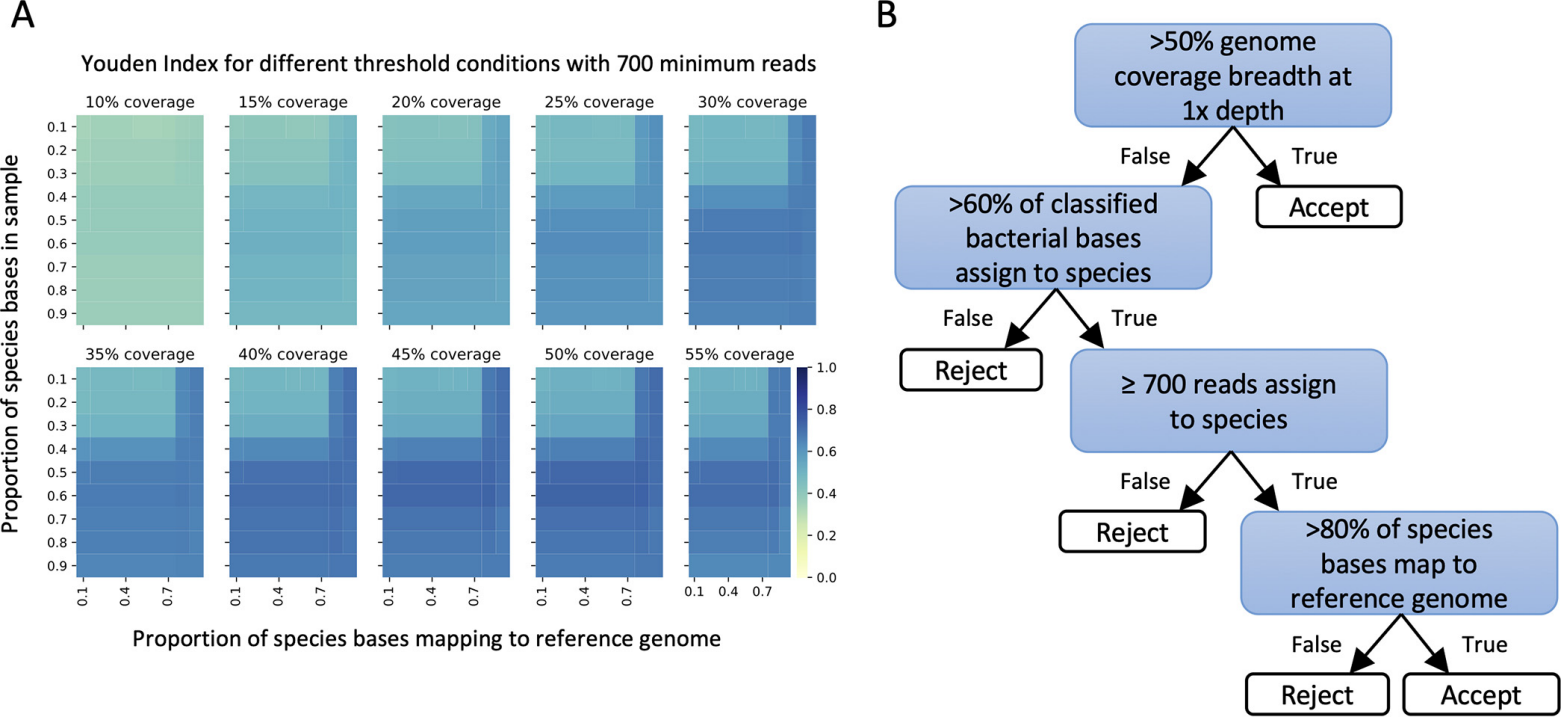
Species detection. Heat map of Youden index (sensitivity + specificity – 1) values at 700 minimum reads per species for each coverage breadth (panels), with proportion of species bases in the sample (*y* axis) over the proportion of these bases that map to a reference genome (*x* axis). Color represents Youden index, where darker blue indicates a more optimal threshold condition (A). Final filtering selection criteria used to determine true presence of a species from metagenomic sequencing reads (B).

Species were identified using metagenomic data from 5% saponin-treated samples. One culture-positive (sample 105, positive for a single organism) failed to generate any sequence data and so was excluded, leaving 114 samples in the analysis. We used filtering to remove taxonomic misclassification and contamination with thresholds determined by maximizing the Youden index (sensitivity + specificity − 1) across a grid search of plausible thresholds (Fig. 2A). We required coverage of >50% of the reference genome or, for samples with less bacterial DNA sequence, >60% of the total bacterial bases from the same species and ≥700 reads from that species with >80% of bases within the reads mapped to the reference genome (Fig. 2B). The requirement for >60% of bacterial bases to be from the same species in low-coverage samples means that some polymicrobial infections may have been missed; however, the majority of species were identified on the basis of the first threshold, i.e., >50% coverage of the reference genome (see below), and cultured polymicrobial infections were uncommon in the study, accounting for 2/115 (2%) of samples.

We used sequencing to attempt to identify 65 individual species from culture-positive sonication fluid samples (50 organisms at >50 CFU/mL, 6 highly pathogenic organisms at <50 CFU/mL and 9 unquantified by culture). Sixty-two (95%) were identified on the basis of >50% coverage of the reference genome and the remaining 3 (5%) by meeting the other thresholds set. Species-level PPA was 50/65 (77%; 95% CI, 65 to 86%) (Table S3). One organism was identified to genus level only by sequencing; hence, the genus-level PPA was 51/65 (78%, 67 to 88%). Of the 14 organisms cultured and not identified by sequencing, 11 were present but below filtering thresholds (3 were cultured at >500 CFU/mL, 4 were cultured at 50 to 500 CFU/mL, 2 were pathogenic organisms at <50 CFU/mL, and 2 were not quantified in culture) and 3 (two Pseudomonas aeruginosa strains and one Candida parapsilosis strain) were not detected in sequence data in samples treated with 5% saponin.

Using sonication fluid culture as the reference standard, NPA was 99/114 (87%; 95% CI, 79 to 92%). However, as some species were found in the tissue samples obtained at the same time, we also calculated NPA using a composite of species found in either the sonication fluid or tissue samples as the reference standard, which was 103/114 (90%; 95% CI, 83 to 95%) (Table S3). Considering only the 48 culture-negative sonication fluids, NPA was 47/48 (98%, 89 to 100%). In 11 samples (including 8 with other species identified), 15 additional species were detected by sequencing. These species included five likely bioinformatic misclassifications leading to identification of an additional species from the same genus: 3 additional Enterobacter cloacae complex species where sonication fluid cultured E. cloacae and one extra *Dermabacter* and Streptococcus species. Five species represented plausible anaerobic infections (Fusobacterium nucleatum, Prevotella intermedia, Cutibacterium acnes, and Anaerococcus mediterraneensis), and two had skin commensal species also cultured in sonication fluid at <50 CFU/mL but not in corresponding PPT (Staphylococcus epidermidis). Three samples had other additional species not otherwise identified by culture (Table S5), including one sample that initially appeared to show potential sample-to-sample cross-contamination, yielding S. aureus in a culture-negative sonication fluid (sample 53) sequenced in the same batch as an S. aureus-positive sample (sample 54). However, the two sequences were >10,000 SNPs different (Fig. S1), suggestive, along with evidence of acute inflammation on histology, of true infection.

In two patients, two separate sonication fluids were cultured from the same surgery. Sequencing matched culture results in both samples for one patient (culture negative and no species detected by sequencing; samples 128a and 128b). For the other patient, although one sonication fluid was culture positive for Streptococcus dysgalactiae and the other negative, 4/5 corresponding PPT samples grew S. dysgalactiae, and both sonication fluid sequences identified S. dysgalactiae (samples 40a and 40b). None of the laboratory negative controls contained species above the filtering thresholds.

### AMR prediction for Staphylococcus aureus.

Sequencing detected S. aureus in 17/19 S. aureus culture-positive sonication fluids and in an additional 2 sonication fluid culture-negative samples with S. aureus culture-positive PPT, totaling 19 samples for AMR prediction. Fourteen of 19 had a single fold genome coverage of >90% (Table 2). For all 19 samples, we compared sequencing-based AMR predictions with phenotypic results for 8 antimicrobials (ciprofloxacin, clindamycin/erythromycin, fusidic acid, methicillin, gentamicin, rifampicin, tetracycline, and trimethoprim).

**TABLE 2:**
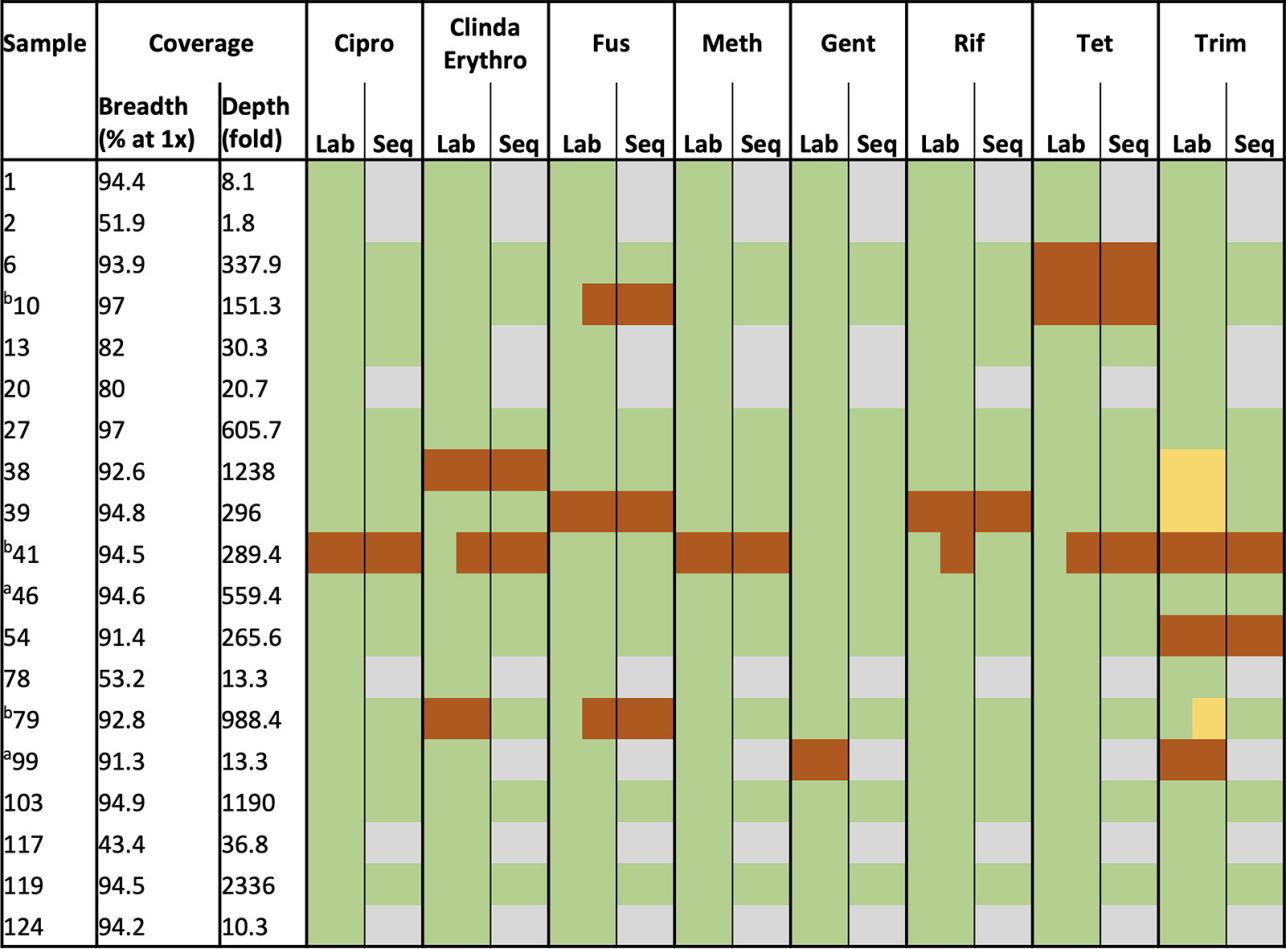
Staphylococcus aureus genome coverage, lab sensitivities, and predicted sensitivities from sequencing^*c*^

a Samples 46 and 99 were culture-negative sonication fluids; results reported here are from corresponding culture-positive PPT for comparison purposes.

b Sensitivities reported for both S. aureus morphotypes cultured in these samples (represented as half green, half orange or yellow).

c Each individual resistance-conferring variant required a coverage depth of at least 20-fold, and resistance genes on mobile genetic elements required at least 20-fold depth plus 100% breadth. Green, susceptible; orange, resistant; yellow, intermediate; gray, unable to determine due to lack of coverage. Cipro, ciprofloxacin; Clinda, clindamycin; Erythro, erythromycin; Fus, fusidic acid; Meth, methicillin; Gent, gentamicin; Rif, rifampicin; Tet, tetracycline; Trim, trimethoprim.

Across 152 organism-drug combinations, sequencing correctly predicted susceptibilities in 87/152 (57%), was unable to call susceptibilities in 60/152 (39%) due to insufficient data, and made an incorrect prediction in 5/152 (3%) (two very major errors [missed resistance] and three minor errors [intermediate phenotype called susceptible]) (Table S6). In three cases, 2 morphotypes were reported with different sensitivities in the laboratory (samples 10, 41, and 79, with different sensitivities for fusidic acid [samples 10 and 79], for trimethoprim [sample 79], and for clindamycin/erythromycin, rifampicin and tetracycline [sample 41]). In these cases of mixed morphotypes, we counted both susceptibilities as a single combination, and sequencing was reported as correct if the resistant genotype was detected.

Sequencing correctly predicted 13/17 (76%) resistance phenotypes reported by the laboratory, with insufficient data for prediction in 2 (12%). Therefore, sequencing correctly predicted 13/15 (87%) of resistance phenotypes where sufficient data were available, with an incorrect result in 2 (13%; described below). Sequencing correctly predicted 74/74 (100%) susceptible phenotypes where sufficient data were available, with insufficient data for 58 susceptible organism-drug combinations. In 3 cases where the laboratory reported an intermediate sensitivity to trimethoprim (samples 38 and 39 and 1 of 2 morphotypes in sample 79), sequencing predicted susceptibility.

In the 8 cases where a successful prediction of antimicrobial susceptibility was made across all drugs, a combination of high genome coverage breadth and depth was observed (Table S6) (>92% breadth at >151-fold depth). Genome breadth or depth of coverage was insufficient in the 5 cases where a partial prediction was made: where breadth was >90%, depth was ≤10-fold, or where depth was >10-fold, breadth was <80%.

We correctly identified the only methicillin-resistant S. aureus isolate (sample 41; 54-fold coverage of *mecA*). Sequencing also correctly identified the only ciprofloxacin-resistant S. aureus isolate (sample 41, *grlA* S80F and *gyrA* S84L). We detected rifampicin resistance in sample 39 (*rpoB* A477V), and for sample 41, which yielded rifampicin-susceptible and -resistant isolates, sequencing detected a C-to-T mutation in 20% of reads mapping to *rpoB*, conferring the A477V substitution (Fig. S2). Our approach, based on the sequence of the majority of reads, called this sample as susceptible, missing the resistant isolate, as our approach does not currently account for mixed populations within the same species.

The second major error identified involved failure to detect any resistance determinants for clindamycin/erythromycin in sample 79, despite high genome coverage (93% at an average depth of 988-fold) and a resistant phenotype for these agents.

Only 3 S. aureus culture-positive samples not treated with saponin prior to DNA extraction (samples 6, 78, and 79) achieved enough breadth and depth of genome coverage to successfully detect any AMR determinants, with predictions made for ciprofloxacin, rifampicin, and fusidic acid only.

## DISCUSSION

Here, we describe the largest study to date of long-read metagenomic sequencing applied to PJI. We show that saponin can effectively deplete human DNA. Using samples treated with saponin, the PPA and NPA for species-level detection were 77% and 90%, respectively. Using Staphylococcus aureus as an exemplar organism, we successfully predicted 13/15 (87%) resistant and 74/74 (100%) susceptible phenotypes where sufficient sequence data were available; 60/152 (39%) drug-organism combinations lacked sufficient data. Five of 152 (3%) yielded incorrect results (three minor errors [intermediate isolates called susceptible] and two very major errors [resistant isolates called susceptible]).

Saponin has previously been shown to reduce host cell contamination in respiratory samples and cerebrospinal fluid (CSF) (17, 20, 27); here, in orthopedic infection-related samples, saponin decreased the proportion of human DNA sequenced from a median of 98% to 12%, causing an increase in bacterial genome coverage and allowing the detection of AMR determinants in S. aureus culture-positive samples. However, 11/49 samples compared achieved incomplete depletion, with a <12% reduction in human bases. Although confirmation of successful human DNA depletion could be performed by quantitative PCR (qPCR), identifying cases where a second depletion might be useful prior to sequencing, this would make workflows more burdensome. We did not record the appearance of each of the sonication fluid samples, but this could be done in future to assess if the presence of visible tissue fragments is associated with incomplete depletion of human DNA.

While saponin treatment enhanced recovery of bacterial DNA overall, it appeared to adversely affect some species. Enterococcus faecalis was not identified by sequencing in one of two culture-positive samples, although it was detected well below the filtering thresholds. The other sample where E. faecalis was detected by sequencing had a paired 5-μm-filtered sample, and here, the number of reads classified as E. faecalis was much higher without saponin treatment. Similar results were observed in 2 of 3 samples culture positive for Pseudomonas aeruginosa, where sequencing reads were detected only in the 5-μm-filtered samples and not after saponin treatment. We hypothesize that sample storage at 4°C between collection and treatment could also have had an adverse effect on the extraction of bacterial DNA from some species. Further work using isolates of these and other bacterial species could determine susceptibility to differing storage conditions and to saponin and other differential lysis methods.

Taxonomic misclassification explained the failure to detect Dermabacter hominis found on culture: sequencing identified the wrong species, Dermabacter vaginalis, as *D. hominis* was not present in the species classification database, reflecting that not all species have available reference genomes at present (but could be available in future). Misclassification of species within the Enterobacter cloacae complex represents a more intrinsic limitation of the method, where individual species may be genetically very similar, analogous to the similar protein composition of some species that makes them difficult to distinguish using MALDI-TOF. We also failed to identify a Candida parapsilosis infection, as the lysis step of our DNA extraction protocol was not designed to disrupt fungi. Sequencing also failed to identify to species level above the filtering thresholds 10 remaining pathogens seen in culture.

Sequencing identified additional species not detected by culture of sonication fluid in 11 cases. In some instances, this may represent taxonomic misclassification of similar species, e.g., within the Enterobacter cloacae complex or between Streptococcus species. We also identified three anaerobic organisms not detected by standard anaerobic culture: Fusobacterium nucleatum, a known cause of PJI (28); Prevotella intermedia, previously reported in a case of osteomyelitis (29); and *Anaerococcus mediterraneensis*. Cutibacterium acnes was identified in two samples. *C. acnes* is a common contaminant but also a well-known cause of PJI. We observed that in *C. acnes* culture-positive samples, the number of bases sequenced, along with genome breadth and depth of coverage, was very high: 100% and 96% breadth with 633-fold and 448-fold depth of coverage, respectively, in the two *C. acnes*-positive samples (samples 12 and 58). One “false-positive” *C. acnes* had a genome coverage breadth and depth of 97% and 384-fold, similar in magnitude to the true positives, i.e., more suggestive of true infection than contamination. This highlights the difficulty of using imperfectly sensitive culture as the reference standard for diagnostic studies, particularly for potentially harder-to-culture organisms. However, we also previously pointed out that contamination with *C. acnes* may also be more common than that with other organisms and that species-specific filtering may be useful (10), but larger sample numbers are required to address this.

The importance of including negative controls for metagenomic sequencing is widely recognized. Laboratory reagents are known to contribute to low-level contamination (30, 31), and previous studies recognized the perils of misinterpreting contamination for true clinical findings (32, 33). In this study, sequencing did not identify any species above the filtering thresholds in any of our 45 negative controls. These findings do depend on filtering our data; while we were able to filter out low-level contaminating DNA and identify most true infections, this likely reduced sensitivity, and further independent studies are needed to validate the filtering thresholds chosen.

In the case of Staphylococcus aureus infection, we were able to successfully predict antimicrobial resistance in 87% and susceptibility in 100% of cases where sufficient sequence data were available. Of the 152 possible organism-drug combinations, insufficient data for prediction were obtained in 39%, highlighting the need for further optimization around laboratory protocols. In 6 cases where two different morphotypes of S. aureus displayed differing sensitivities, sequencing was able to successfully predict resistance in 4 cases. The fifth case, with both sensitive and intermediate susceptibility to trimethoprim, was reported by sequencing as susceptible (as were 2 other samples that were reported with an intermediate phenotype). Resistance determinants were detected in a proportion of sequence reads in the sixth case, serving to emphasize the need for bioinformatic methods that account for mixed infections, which our current approach does not. Long sequence reads provide more genetic context, allowing the assignment of chromosomal determinants to species fairly accurately (16), but AMR determinants carried on plasmids present more of a challenge.

Our work adds to other recent reports. Wang et al. (13) assessed nanopore sequencing for diagnosis of PJI, identifying the cause of infection in 4/5 sonication fluid samples. A small number of AMR determinants were identified in 2/5 samples, although concordance with laboratory sensitivities was variable. Yan et al. (34) previously assessed a commercial metagenomic data analysis service for the detection of antimicrobial resistance determinants specifically in staphylococcal species from sonication fluid and reported sensitivities ranging from 66 to 85%, similar to the 87% we observed in this study. Metagenomic sequencing for AMR determinant detection has also been applied to PPT samples for bone and joint infections (9), with the correct susceptibility inferred in 94.1% of monomicrobial and 76.5% of polymicrobial samples. Recent reviews also highlight the use of metagenomic sequencing for AMR detection (35, 36).

Limitations of metagenomic sequencing, observed both here and in previous studies (for example, see references 11 and 37), include the need for each species to be present in the reference database and also the need for unbiased panbacterial/panfungal species-agnostic DNA extraction (e.g., here, we failed to detect a *Candida* species on this basis). The need to filter data means that low-level and polymicrobial infections may be erroneously filtered out and missed. Using machine learning with a properly balanced training data set to determine thresholds for true positives may be helpful, particularly when considering polymicrobial infections, but would require a higher number of these than the current sample set allows. Finally, successful AMR prediction relies on both the range of resistance determinants present in the reference catalogue and obtaining a combination of high genome coverage breadth and depth, which is directly linked to the proportion of human DNA sequenced. We did not have sufficient remaining DNA to repeat sequencing for samples with too few data. We also predicted antimicrobial susceptibilities only for S. aureus; further work is required to develop algorithms and databases for prediction in other organisms that can account for resistance arising from chromosomal mutations, mobile genetic elements, and changes in gene copy number.

In conclusion, metagenomic sequencing is a useful addition to the repertoire of diagnostic tests performed in the case of suspected PJI. Although sensitivity remains below that which would be needed to replace culture as a gold standard diagnostic method, the detection of additional pathogens is useful in culture-negative samples. Saponin is a useful method for host DNA depletion, but adverse effects on some pathogens mean that better approaches to human DNA depletion that preserve all microbial DNA are required. Additionally, a microbial DNA extraction method that encompasses all pathogens is necessary so that fungal infections can be detected in addition to bacterial ones. Our study has demonstrated as a proof of principle that nanopore sequencing in conjunction with human DNA depletion shows potential for the detection of AMR determinants using S. aureus as a model organism for PJI. Further development is required to test this method on a wider range of pathogens.
