# Supplementary material for: Clinical Metagenomic Sequencing for Species Identification and Antimicrobial Resistance Prediction in Orthopedic Device Infection
Source: J Clin Microbiol. 2022 Mar 31;60(4):e02156-21. doi: 10.1128/jcm.02156-21 (PMC9020354; doi:10.1128/jcm.02156-21)
Supplement: Supplemental file 1 — Supplemental methods, Fig. S1 and S2, and Tables S1, S2, and S4 to S6. Download jcm.02156-21-s0002.pdf, PDF file, 1.0 MB [file jcm.02156-21-s0002.pdf]

Supplementary Material

Supplementary Methods

Human DNA depletion and bacterial DNA extraction

We tested the impact of host cell DNA depletion with saponin, using the method outlined by Charalampous et al[1]. Prior to human DNA depletion and bacterial DNA extraction, 40 ml of fresh sonication fluid, stored at 4°C between generation and use, was concentrated by centrifugation as previously described[2], yielding an initial volume of 1-2 ml. After further centrifugation at 8000 g for 5 minutes, the pellet was resuspended in 250 µl of PBS and 200 µl of 5% saponin solution was added, giving a final concentration of 2.2% in each depletion reaction. As a control, an equal volume of sonication fluid was concentrated by centrifugation and passed through a 5 µm syringe filter instead of treatment with saponin. After host DNA depletion, bacterial DNA was extracted by mechanical lysis followed by ethanol precipitation, as previously described[2]. DNA was purified using AMPure XP solid phase reversible immobilisation (SPRI) beads (Beckman Coulter, High Wycombe, UK) and eluted in a final volume of 26 µl TE buffer. Negative controls for sonication (0.9% saline) and before saponin treatment (PBS) or 5 µm filtration (0.9% saline) were prepared alongside the extractions from sonication fluids.

Library preparation and sequencing

DNA extracts were prepared for sequencing on an Oxford Nanopore Technologies (ONT, Oxford, UK) GridION using the Rapid PCR Barcoding Kit (SQK-RPB004, ONT) and a modified protocol as previously described[3]. Initially, PCR libraries were purified individually with AMPure XP beads and eluted in a total volume of 10 µl before quantification on a Qubit 2.0 fluorimeter with the Quant-iT dsDNA HS Assay kit (Life Technologies, Paisley, UK). Purified libraries were multiplexed up to a maximum of 8, with a total of between and 25 and 302 fmol of pooled library loaded per flow cell, for the first 11 flow cells. Subsequent libraries were quantified immediately post-PCR, pooled together by similar concentrations

then purified with AMPure XP beads and eluted in 10 µl. Here, a total of between 6 and 84 fmol of pooled library, corresponding to a maximum of 8 multiplexed libraries per flow cell, were loaded for all subsequent flow cells.

Nanopore sequence processing and analysis

Nanopore sequences were basecalled and demultiplexed using Guppy (Oxford Nanopore Technologies, Version 3.1 or higher) automatically on the GridION platform. Sequences were analysed using our in-house workflow CRuMPIT, described previously[4]. Briefly, sequences are classified with Centrifuge[5], binned into species-specific groups and aligned to a reference genome for that species. Reads classified as human are discarded and reads classified to a lower resolution taxon than species, e.g. genus only, are not mapped. Some runs were performed prior to Guppy providing demultiplexing by default on the GridIONs and in these cases Porechop v0.2.4[6] was used for demultiplexing. The fastq files from these runs had adapters trimmed and therefore could not be demultiplexed again with Guppy, so here we basecalled from fast5 files and then demultiplexed these runs with Guppy to maintain consistency with later runs. Human reads had, however, already been discarded from these fast5 files to comply with ethical requirements, so human read number comparisons use a mix of results from either Porechop or Guppy. Since comparisons are between treatments of the same sample sequenced on the same run the analysis should not be affected.

Determining species detection performance

Species classifications from Centrifuge and CRuMPIT mapping metrics, including coverage breadth and proportion of bases mapping to the reference, were compared to standard microbiological culture results. We used species presence in sonication fluid culture at >50 CFU/ml (or ≤50 CFU/ml of a highly pathogenic organism) as the reference standard for presence of a bacterial species. When evaluating negative percent agreement (NPA), we performed two analyses. In the first all species not present in the sonication fluid cultures were considered false positives. However, as sonication fluid culture is an imperfect

reference standard, in a second analysis species present in PPT cultures and not sonication fluid cultures, were not considered false-positive results. We did not apply this second composite reference standard to positive percent agreement (PPA) calculations as for anatomical reasons it is possible that some species identified in PPT cultures were never present in the sonication fluid, and therefore could not be sequenced.

Where standard culture was reported to the genus level only, species belonging to the same genus were counted as a match. In this setting, multiple species matching to one genus report were reduced to a single match to avoid artificially inflated PPA results.

Species detection filtering

To distinguish between true species classified by Centrifuge and misclassifications or low-level contamination, filtering thresholds were determined. We used three metrics for filtering. The first used the percentage of the identified species reference genome covered by sequence reads. Secondly, if the sample had low overall numbers of bacterial reads with no species identified above the given percent coverage, the proportion of bases classified as a species compared to total bacterial bases in the sample, along with the number of reads assigned to that species, was used. Thirdly, as reads can be classified with lower specificity by Centrifuge to improve sensitivity, the proportion of species bases mapping to the reference genome was also considered. Thresholds for each filter were determined by choosing the combination of thresholds that maximised the Youden index (specificity+sensitivity-1).

References

1. Charalampous T, Kay GL, Richardson H, et al. Nanopore metagenomics enables rapid clinical diagnosis of bacterial lower respiratory infection. Nat Biotechnol **2019**; 37:783–792.

2. Street TL, Sanderson ND, Atkins BL, et al. Molecular Diagnosis of OrthopedicDevice-Related Infection Directly from Sonication Fluid by Metagenomic Sequencing. J Clin Microbiol **2017**; :JCM.00462-17.

3. Street TL, Barker L, Sanderson ND, et al. Optimizing DNA Extraction Methods for Nanopore Sequencing of Neisseria gonorrhoeae Directly from Urine Samples. J Clin Microbiol **2020**; 58. Available at: https://jcm.asm.org/content/58/3/e01822-19. Accessed 3 July 2020.

4. Sanderson ND, Street TL, Foster D, et al. Real-time analysis of nanopore-based metagenomic sequencing from infected orthopaedic devices. BMC Genomics **2018**; 19:714.

5. Kim D, Song L, Breitwieser FP, Salzberg SL. Centrifuge: Rapid and sensitive classification of metagenomic sequences. Genome Res **2016**; 26:1721–1729.

6. Wick RR. Porechop. 2018. Available at: https://github.com/rrwick/Porechop.

7. Gordon NC, Price JR, Cole K, et al. Prediction of Staphylococcus aureus Antimicrobial Resistance by Whole-Genome Sequencing. J Clin Microbiol **2014**; 52:1182–1191.

Supplementary Data

Supplementary Figures

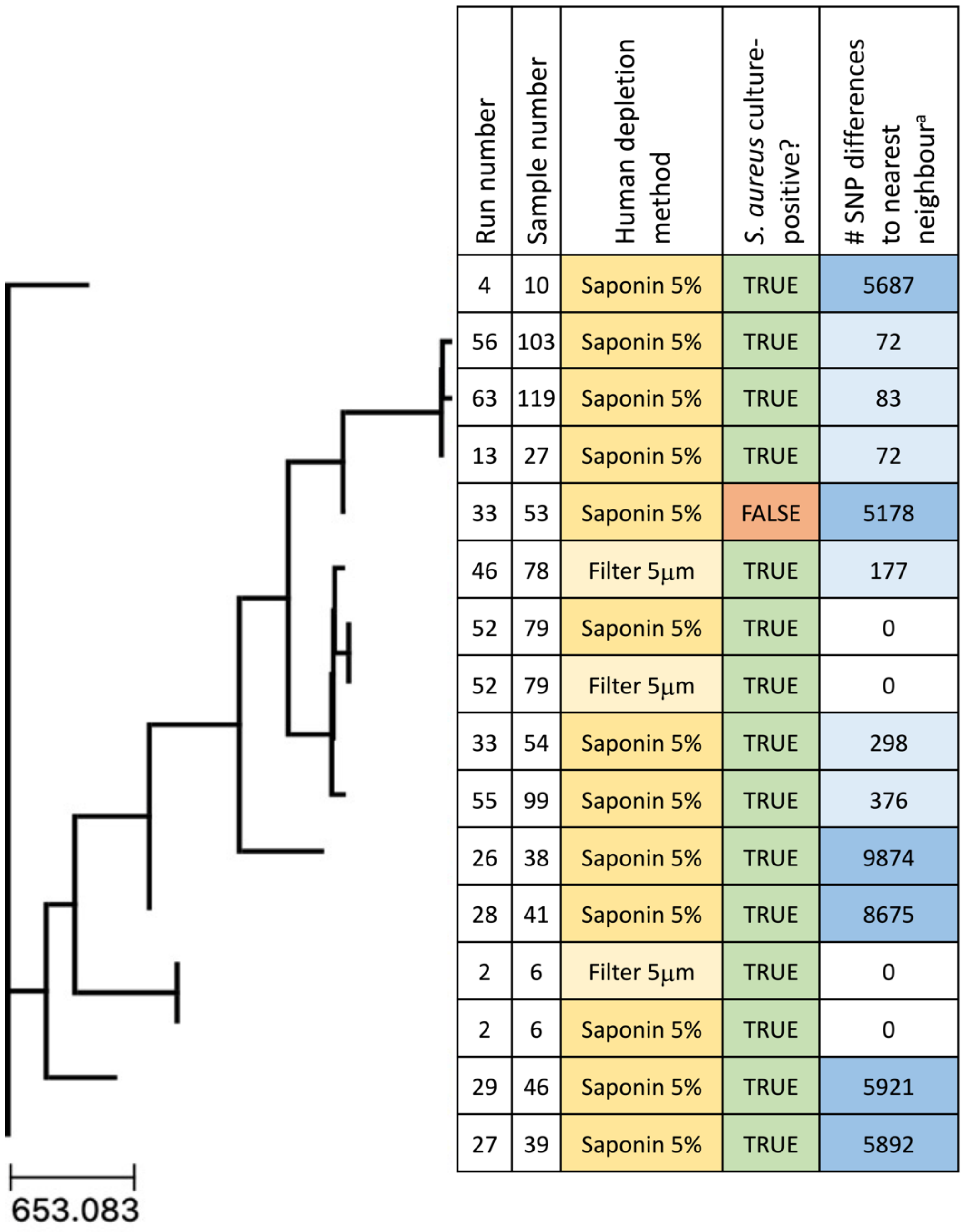

Run number	Sample number	Human depletion method	*S. aureus* culture-positive?	# SNP differences to nearest neighbour[a]
4	10	Saponin 5%	TRUE	5687
56	103	Saponin 5%	TRUE	72
63	119	Saponin 5%	TRUE	83
13	27	Saponin 5%	TRUE	72
33	53	Saponin 5%	FALSE	5178
46	78	Filter 5μm	TRUE	177
52	79	Saponin 5%	TRUE	0
52	79	Filter 5μm	TRUE	0
33	54	Saponin 5%	TRUE	298
55	99	Saponin 5%	TRUE	376
26	38	Saponin 5%	TRUE	9874
28	41	Saponin 5%	TRUE	8675
2	6	Filter 5μm	TRUE	0
2	6	Saponin 5%	TRUE	0
29	46	Saponin 5%	TRUE	5921
27	39	Saponin 5%	TRUE	5892

Figure S1. Phylogenetic tree of Staphylococcus aureus genome sequences. *There are >10k SNPs between sequences for sample 53 and 54, and over 5k SNPs between sample 53 and other samples sequenced in the lab. [a]Dark blue represents SNP differences >5000, light blue represents SNP differences <5000.*

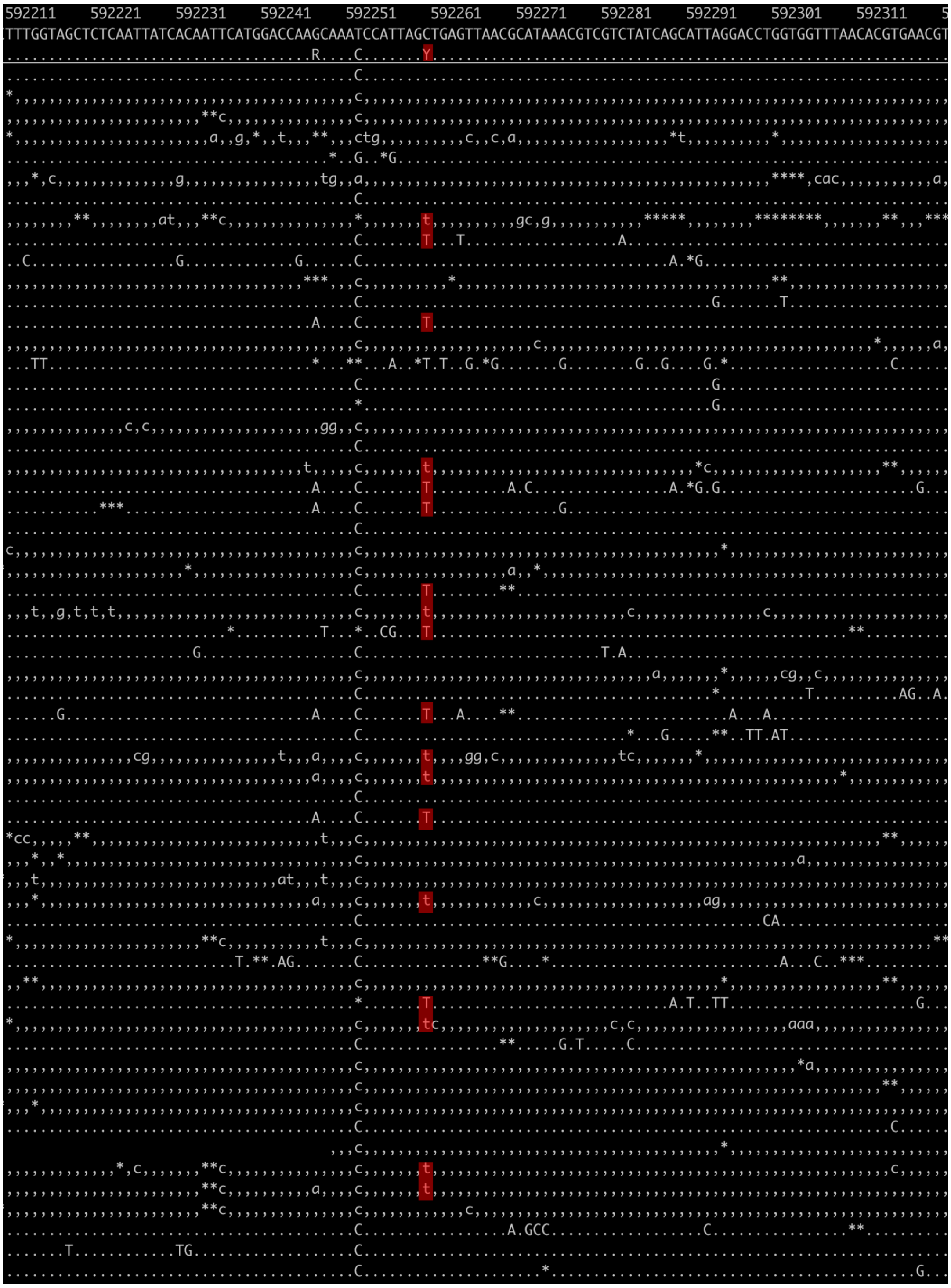

Figure S2. rpoB mutation observed in sample 41 at position 592260 in the reference genome.

Nucleotide mutation C to T causing amino acid codon substitution from GCT (A) to GTT (V) at residue 477, conferring resistance to rifampicin, highlighted in red. T substitution observed in 20% of reads aligning to this position.

Supplementary Tables

**Antimicrobial agent**	**Gene**	**Amino acid substitutions**	**Reference gene accession no. (nucleotide positions)**
Ciprofloxacin	*gyrA*	S84L, E88K, G106D, S85P, E88G, E88L	BX571857.1 (7005–9668)
	grlA	S80F, S80Y, E84K, E84G, E84V, D432G, Y83N, A116E, A48T, D79V, V41G, S108N	BX571857.1 (1386869–1389271)
	grlB	R470D*, E422D*, P451S*, P585S*, D443E*, R444S*	BX571857.1 (1384872–1386869)
Fusidic acid	*fusA*	A160V*, A376V, A655E, A655P*, A655V*, A67T*, A70V*, A71V*, B434N, C473S*, D189G*, D189V*, D373N*, D463G*, E233Q*, E444K, E444V*, E449K*, F441Y, F652S*, G451V, G452C, G452S, G556S, G617D, G664S, H438N, H457Q, H457Y, L430S*, L456F, L461K, L461S, M161I*, M453I, M651I, P114H, P404L, P404Q, P406L, P478S, Q115L, R464C, R464H, R464S, R659C, R659H, R659L, R659S, R76C*, S416F*, T385N, T387I*, T436I, T656K, V607I, V90A, V90I, Y654N*	BX571857.1 (577685–579766)
Rifampicin	*rpoB*	A473T*, A477D, A477T*, A477V, D471G*, D471Y, D550G, H481D, H481N, H481Y, I527F, I527L*, I527 M*, ins 475H, ins G475*, L466S*, M470T*, N474K*, Q456K, Q468K, Q468L, Q468R, Q565R*, R484H, S463P, S464P, S486L, S529L*	BX571857 (568813–572364)
Trimethoprim	*dfrB*	F99Y, F99S, F99I, H31N, L41F, H150R, L21V*, N60I*	BX571857.1 (1464014–1464493)

Table S1. Polymorphisms conferring resistance in chromosomal genes. *Asterisk (*) represent mutations in combination. Adapted from Gordon* et al.*[7]*

Antimicrobial agent(s)	Gene	Product	Reference gene accession no. (nucleotide positions)
Methicillin	*mecA*	Low-affinity PBP2	BX571856.1 (44919–46925)
Erythromycin	*msrA*	Erythromycin resistance protein	CP003194 (54168–55634)
Erythromycin and clindamycin	*ermA*	rRNA adenine N-6-methyltransferase	BA000018.3 (56002–56733)
	ermB	rRNA adenine N-6-methyltransferase	AB699882.1 (4971–5708)
	ermC	rRNA adenine N-6-methyltransferase	HE579068 (7858–8592)
	ermT	23S rRNA methylase	HF583292 (11344–12078)
	lsaE	ATP-binding cassette (ABC) ribosomal protection protein, family F	JX560992 (11387-12872)
	vgaE	ATP-binding cassette (ABC) ribosomal protection protein, family F	FR772051 (8740-10315)
	lnuA	Lincosamide nucleotidyltransferase	AM399080 (1664-2150)
	lnuB	Lincosamide nucleotidyltransferase	AY183453.1 (2730-3950)
	ereA	Erythromycin esterase	X03988.1 (383-1642
	ereB	Erythromycin esterase	AE007317.1 (383-1642)
	mefE	Macrolide efflux pump	FJ196385.1 (11084-12313)
	mefB	Macrolide efflux pump	AB571865.1 (144313-145536)
	mefC	Macrolide efflux pump	MN728681.1 (17459-18658)
	mefD	Macrolide efflux pump	AB013298.1 (2296-3195)
	mphC	Macrolide phosphotransferase	AJ238249.1 (127-930)
Tetracycline	*tetK*	MFS tetracycline efflux pump	FN433596 (69118–70497)
	tetL	MFS tetracycline efflux pump	HF583292 (7713–9089)
	tetM	Ribosomal protection protein	CP002643 (427033–428952)
Fusidic acid	*fusB*	Fusidic acid detoxification	CP003193.1 (1336–1977)
	fusC	Fusidic acid detoxification	BX571857.1 (52820-53458)
	far	Ribosome protection protein	AY373761.1 (19072–19713)
Trimethoprim	*dfrA*	Insensitive dihydrofolate reductase	CP002120 (2093303–2093788)
Trimethoprim	*dfrG*	Insensitive dihydrofolate reductase	FN433596 (502263–502760)
Gentamicin	*aacA-aphD*	6′-aminoglycoside N-acetyltransferase/2″-aminoglycoside phosphotransferase	FN433596.1 (2209531–2210970)

Table S2. Mobile resistance genes. *Presence of these genes is associated with resistance to the respective antibiotic. Adapted from Gordon* et al.*[7]*

Sample	Saponin human proportion	Filter human proportion	% of original human proportion	% reduction in human proportion of bases
1	0.97	0.98	98.66	1.34
6	0.00	0.72	0.28	99.72
8	0.99	0.99	99.82	0.18
9	0.03	1.00	2.85	97.15
10	0.01	0.99	1.29	98.71
12	0.13	0.98	13.19	86.81
18	0.01	0.93	0.60	99.40
20	0.00	0.74	0.16	99.84
22	0.00	0.70	0.51	99.49
26	0.32	0.98	32.51	67.49
31	0.89	1.00	88.98	11.02
33	0.00	0.96	0.29	99.71
34	0.67	1.00	67.10	32.90
38	0.00	0.99	0.46	99.54
39	0.04	1.00	3.67	96.33
41	0.00	0.76	0.12	99.88
42	0.16	0.85	18.68	81.32
43	0.01	0.36	2.00	98.00
45	0.18	0.99	17.65	82.35
54	0.03	0.98	3.23	96.77
55	0.12	1.00	11.95	88.05
56	0.89	0.99	90.20	9.80
57	0.49	1.00	49.49	50.51
58	0.04	0.76	5.10	94.90
59	0.21	0.98	21.41	78.59
60	0.00	0.64	0.46	99.54
61	0.00	0.56	0.12	99.88
62	0.16	1.00	16.27	83.73
63	0.00	0.54	0.24	99.76
66	0.36	1.00	35.99	64.01
72	0.00	0.97	0.03	99.97
74	0.44	1.00	43.95	56.05
75	0.72	0.99	73.10	26.90
78	0.11	0.97	11.51	88.49
79	0.00	0.87	0.10	99.90
81	0.00	0.53	0.12	99.88
87	0.92	1.00	92.61	7.39
90	1.00	1.00	99.93	0.07
96	0.96	1.00	96.15	3.85
100	0.86	0.94	91.84	8.16
103	0.01	0.99	1.16	98.84

104	0.06	0.98	5.94	94.06
109	0.95	1.00	95.17	4.83
112	0.00	0.90	0.19	99.81
115	0.22	0.30	75.68	24.32
116	0.01	0.98	0.89	99.11
117	0.16	0.99	16.04	83.96
120	0.92	0.98	93.38	6.62
126	0.97	0.98	98.35	1.65

Table S4. Effect of 5% saponin treatment on proportion of human bases sequenced. Percent reduction in proportion of human bases sequenced following saponin treatment in comparison to 5µm filter treatment. 100% reduction means all human bases removed.

Sample	False-positive species	Evidence of acute inflammation on histology?	Ref. genome coverage (%)	Average depth (fold)	Interpretation
3	*Enterobacter ludwigii*	Yes	54	3	[a]*Enterobacter cloacae* complex species
	Cutibacterium acnes		59	2	Plausible anaerobic pathogen/skin flora contamination
20	*Fusobacterium nucleatum*	Yes	79	363	Plausible anaerobic pathogen
25	*Enterobacter hormaechei*	Yes	92	628	[a]*Enterobacter cloacae* complex species
53	*Staphylococcus aureus*	Yes	89	6	Potential sample-to-sample contamination/plausible infection
59	*Corynebacterium segmentosum*	Yes	74	38	Skin flora contamination/plausible infection
81	*Streptococcus* sp. NPS 308	Yes	67	121	Correct to genus level/[b]misclassification
99	*Dermabacter vaginalis*	Yes	58	2.5	Correct to genus level
	Anaerococcus mediterraneensis		62	100	[b]Misclassification/plausible anaerobe
	Prevotella intermedia		90	40	Plausible anaerobe
	Enterobacter hormaechei		87	26	[a]*Enterobacter cloacae* complex species
109	*Staphylococcus epidermidis*	Yes	93	52	Cultured at <50 CFU/ml in sonication fluid
112	*Cutibacterium acnes*	Yes	97	383	Plausible anaerobic pathogen/skin flora contamination
116	*Staphylococcus epidermidis*	Yes	78	15	Cultured at <50 CFU/ml in sonication fluid
120	*Corynebacterium segmentosum*	No	72	11	Skin flora contamination/plausible infection

Table S5. Additional species identified. *Summary of potential 'false-positive' species identified by metagenomic sequencing, showing percentage of reference genome mapped and the average fold-depth of coverage. [a]Enterobacter cloacae complex species, indicates where E. cloacae was observed by culture in these samples; [b]misclassification, suggestive of bioinformatic misclassification to a different species of the same genus.*

Sample	% coverage of mobile gene				Fold-coverage of chromosomal genes								Detected gene mutations in chromosomal genes					
	ermA	*ermC*	*fusC*	*tetK*	*blaZ*	*dfrB*	*fusA*	*grlA*	*grlB*	*gyrA*	*mecA*	*rpoB*	*dfrB*	*fusA*	*grlA*	*grlB*	*gyrA*	*rpoB*
1	N	N	N	N	56.2	3.6	12.9	5.3	7.9	13.2	N	9.2	N	B434D, E449I, F441T, M161P, T387D	N	R470F, E422N, P451I, P585M, D443G	N	Q456S, Q565A
6	N	N	N	100	1122.4	90.2	83.5	144.9	166.8	124.3	N	89.0	N	B434D, E449I, F441T, M161P, T387D	N	R470F, E422N, P451I, P585M, D443G	N	Q456S, Q565A
10	N	N	100	100	326.6	45.6	100.2	80.9	96.3	120.9	N	129.0	N	B434D, E449I, F441T, M161P, T387D	N	R470F, E422N, P451I, P585M, D443G	N	Q456S, Q565A
13	N	N	N	N	141.6	2.0	34.2	83.0	51.1	15.6	N	23.1	N	B434D, E449I, F441T, M161P, T387D	N	R470F, E422N, P451I, P585M, D443G	N	Q456S, Q565A
20	N	N	N	N	N	N	N	N	N	N	N	N	N	N	N	N	N	N
27	N	N	N	N	48.1	88.6	101.3	96.3	78.1	142.4	N	72.2	N	B434D, E449I, F441T, M161P, T387D	N	R470F, E422N, P451I, P585M, D443G	N	Q456S, Q565A
38	100	N	N	N	N	93.9	91.9	95.7	92.6	136.3	N	87.3	N	B434D, E449I, F441T, M161P, T387D	N	R470F, E422N, P451I, P585M, D443G	N	Q456S, Q565A
39	N	N	100	N	422.5	119.2	75.0	122.1	110.7	77.5	N	173.1	N	B434D, E449I, F441T, M161P, T387D	N	R470F, E422N, P451I, P585M, D443G	N	A477V, Q456S, Q565A
41	N	100	N	100	78.5	79.6	104.5	98.4	96.8	102.7	54.4	92.4	L21V	B434D, E449I, F441T, M161P, T387D	S80F	R470F, E422N, P451I, P585M, D443G	S84L	Q456S, Q565A
46	N	N	N	N	122.0	90.9	104.9	93.9	102.2	98.3	N	107.0	N	B434D, E449I, F441T, M161P, T387D	N	R470F, E422N, P451I, P585M, D443G	N	Q456S, Q565A
54	N	N	N	N	N	182.9	89.8	57.9	77.0	45.6	N	189.3	F99Y	B434D, E449I, F441T, M161P, T387D	N	R470F, E422N, P451I, P585M, D443G	N	Q456S, Q565A
78	N	N	N	N	N	N	N	N	N	N	N	N	N	N	N	N	N	N
79	N	N	N	N	900.4	90.1	102.5	96.6	99.9	114.1	N	106.8	N	B434D, E449I, F441T, M161P, T387D, V90I	N	R470F, E422N, P451I, P585M, D443G	N	Q456S, Q565A
99	N	N	N	N	4.9	27.2	19.3	11.7	7.8	12.7	1.0	10.8	N	B434D, E449I, F441T, M161P, T387D	N	R470F, E422N, P451I, P585M, D443G	N	Q456S, Q565A
103	N	N	N	N	75.7	93.9	89.3	117.9	99.5	117.3	N	79.9	N	B434D, E449I, F441T, M161P, T387D	N	R470F, E422N, P451I, P585M, D443G	N	Q456S, Q565A
117	N	N	N	N	N	N	N	N	N	N	N	N	N	N	N	N	N	N
119	N	74.7	N	N	45.1	101.5	94.8	95.4	98.2	117.8	N	90.6	N	B434D, E449I, F441T, M161P, T387D	N	R470F, E422N, P451I, P585M, D443G	N	Q456S, Q565A
124	N	N	N	N	N	15.0	7.9	5.8	4.9	12.5	N	9.4	N	B434D, E449I, F441T, M161P, T387D	N	R470F, E422N, P451I, P585M, D443G	N	Q456S, Q565A

Table S6. Sequence information for antimicrobial resistance determinants. Percent coverage of mobile resistance genes where detected, fold-coverage of chromosomal genes and detected mutations in chromosomal genes. Only genes with mapped sequence data are reported.
